# Predictors for runaway behavior in adolescents in South Korea: national data from a comprehensive survey of adolescents

**DOI:** 10.3389/fpsyt.2023.1195378

**Published:** 2023-08-16

**Authors:** Hyung Ran Kim, So-Hyun Moon

**Affiliations:** ^1^Department of Health Insurance Review and Assessment Service, Kwangju Christian Hospital, Gwangju, Republic of Korea; ^2^Department of Nursing, College of Medicine, Chosun University, Gwangju, Republic of Korea

**Keywords:** adolescent, runaway behavior, media, family relations, ecological

## Abstract

**Background:**

Runaway behavior is reported to impede the growth, mental health development, and social adjustment of adolescents. Exposure to harmful media causes problematic behaviors in adolescents, sometimes inducing them to run away from home.

**Methods:**

This study examined the factors influencing adolescents’ runaway behavior. Utilizing the data of 11,354 adolescents from the Survey of Media Usage and Harmful Environment among Adolescents, a hierarchical logistic regression analysis was conducted using the SPSS 24.0 program.

**Results:**

The significant predictors of runaway behavior were the grade of the adolescent, deviant behaviors (drinking, smoking), autonomous control ability, relationship with family, and harmful media (*p* < 0.001). This regression model explained 13.1% of the variance in runaway behavior. A significant outcome of this study is that harmful media was identified as one of the factors affecting adolescents’ runaway behavior. Adjusted OR and 95% CI of harmful media was 1.23 (1.10–1.38).

**Conclusion:**

This study showed that individual, family, social factors, and harmful media influence adolescents’ runaway behavior. The results emphasize the importance of health teachers and the need for early intervention programs, for the identification and prevention of risk factors for adolescents’ runaway behavior.

## 1. Introduction

### 1.1. Tendencies and characteristics of south Korean adolescent runaways

In 2021, 3.2% of adolescents said they had run away from home in the past year, an increase of 0.3% compared to the previous year. The percentages of adolescents who had run away from home in the previous year were 2.7% of middle school students, 2.6% of high school students, and 2.1% of elementary school students in South Korea (1).

By age, 55.5% of adolescents run away from home between the ages of 13 and 15 (1), and the number of runaways among elementary school students is increasing. This reflects that runaway behavior is occurring at a younger age (1–4).

Adolescence is a critical transitional period where one establishes their ego-identity and undergoes rapid changes in one’s body, cognition, emotion, morality, and social identity (5, 6). Overall, individuals tend to engage in more risky behaviors in adolescence than in any other developmental stage (7). Runaway adolescents are exposed to alcohol and drugs (8–10) and exhibit psychological problems such as depression (11), self-injurious behavior (12), and suicidal tendencies (8, 10, 13). They are also introduced to many developing antisocial and delinquent behavior such as dropping out of school, theft for a living, rape, prostitution (6, 13, 14), and physical violence (15, 16). Runaway behavior serves as a major variable that interferes with growth, development of mental health, and social adaptation in adolescents (5, 13). As runaway behavior is emerging as a serious social problem that adversely affects adolescents and the community, active intervention is needed (4). A harmful environment disrupts the mature and healthy development of adolescents (6), influencing their delinquent behavior (4, 17). Recently, the types of harmful environments have diversified and become highly accessible (6), which has led to adolescents being increasingly exposed to them while they spend time with their peers (18, 19). Exposure to a harmful environment may serve as a mediator of adolescents’ delinquency behavior (18), and the experience of adolescents’ visits to harmful facilities such as karaoke bars, pubs, nightclubs, and video rooms, is emerging as a social problem encouraging them to run away from home (17, 20). Harmful media for juveniles are media such as movies, videos, adult gaming, music, performances, the internet, publications, and advertisements that contain sexually suggestive and violent content harmful to young people and are therefore inappropriate for distribution to young people (21).

In 2021, 4 out of 10 teenagers (37.0%) were at risk of over-dependence on smartphones, and the risk group for over-dependence on smartphones increased by 1.2% points year-on-year (1).

The increase in smartphone usage is drawing serious attention as a factor contributing to increased exposure to harmful media (2) as 76.1% of adolescents were using the internet and mobile messenger apps almost daily, and 95% were using smartphones for exposure to media such as adult videos or magazines, and adult online games (22). These results indicate an increasing risk of adolescents’ exposure to harmful internet content through excessive use of smartphones (1, 22).

The technology-driven social structure also provided new models and opportunities for teenagers to form “runaway fams.” They live in groups and call themselves a “runaway fam.” These “runaway fams” are associated with group crimes committed by organized, intelligent, and cruel adolescents in South Korea (23), causing complicated social problems.

### 1.2. Predictors of runaway behavior among youth

The factors influencing adolescents’ runaway behavior are discussed from an individual, family, and social environmental perspective: individual factors, such as being female (22, 24, 25), gender (26, 27), being 15 years of age or older (28), types and grade levels in school (29), and ego identity (5); family factors such as single-parent family (3, 30), conflict with parents (11, 31), parental attachment (14), and physical abuse (25, 26); and environmental factors such as academic performance at school (4, 25, 31), being a victim of violence at school (24, 26, 30), and the local community one resides in Moon, Cauffman et al., and Heerde et al. (3, 20, 31). These problems are influenced not by a single factor such as school, family, or individual but by a combination of these within the environment (13). To understand this, an approach with an ecological system perspective that focuses on explaining the process of individuals maintaining dynamic balance or undergoing changes, while influencing one another through ongoing social interaction (14, 25), is required.

A considerable amount of research on adolescents has integrated a risk factor approach to Bronfenbrenner’s ecological systems framework (32, 33) to identify multiple risk factors that increase youths’ vulnerability and susceptibility to negative developmental outcomes.

The ecological perspective focuses on explaining the process of maintaining or changing the dynamic equilibrium, influencing individuals through interactions with each other while living in a particular environment (14, 34). According to the ecological approach, adolescents grow and develop within diverse and complicated socio-environmental systems, emphasizing the importance of the environment as a part of this system for youth (26). An integrated ecological framework of risk factors for runaway behavior would suggest that multiple risk factors are related to runaway behavior and that these factors are “nested” and operate at multiple levels, including the individual (e.g., sociodemographic factors, child abuse, substance abuse), familial (e.g., family instability), and extra-familial levels (e.g., school factors, peer networks) (35–39).

Gottfredson and Hirschi’s theory of low self-control has generated a considerable amount of research and the results of these studies have shown that low levels of self-control are consistently associated with involvement in antisocial outcomes (40). The current study examines the efficacy of low self-control in predicting the involvement of South Korean adolescents in typical delinquency, drinking, smoking, Internet addiction, and smartphone addiction (41). Autonomy is regarded as one of the basic psychological needs that contribute to adaptive psychosocial functions. Such a psychological need is particularly highlighted in adolescence due to the increased demand for autonomy-seeking during this period (42).

According to Gottfredson and Hirschi’s Generality Hypothesis, people who lack self-control are risk taking and they are also more likely to experience problems in social relationships, such as drug and alcohol abuse. They also argue that the cause of low self-control lies with parents and that parents should be able to monitor their children, recognize bad behaviors, and correct these bad behaviors. Based on this, it is necessary to comprehensively examine the self-control ability and the relationship with parents as influencing factors for adolescents running away from home. Previous studies analyzing the correlation between adolescents’ runaway behavior and individual (self-esteem), family (conflicts or support), and school factors (teacher support) (3), as well as other influencing factors, have limitations in only elucidating unilinear relationships among the variables.

Studies investigating influencing factors for runaway behavior (25, 26) only examined the current status of runaways. Only a few studies have comprehensively investigated the cause of adolescents’ runaway behavior by studying environmental factors such as harmful media that can affect adolescents, in addition to individual factors based on the current status of runaways.

To understand adolescents’ runaway behavior, it is important to understand its risk factors early and to proactively intervene and prevent them rather than prepare countermeasures. Therefore, in this study, a multi-level analysis was done of the relationships between runaway experience and individual, family, and social factors; harmful media; and other influencing factors. The analysis is based on the raw statistics data from the “Comprehensive Survey of Adolescents’ Contact with Media Usage and Harmful Environment” to provide basic data for preparing coping measures and programs that can reduce runaway behavior in adolescents.

### 1.3. The purpose of the study

This study aims to understand the factors influencing the runaway experience in Korean adolescents.

First, it examines the differences in general characteristics of adolescents according to the presence of runaway experience. Second, it examines the differences in runaway frequency according to general characteristics of adolescents and differences in related variables according to the presence of runaway experience. Third, the factors associated with adolescents’ runaway experience are determined.

## 2. Materials and methods

### 2.1. Data collection

This study is a secondary data analysis of the 2016 Comprehensive Survey of Adolescents’ Contact with Media Usage and Harmful Environment (2). The survey was conducted by the Ministry of Gender Equality and Family and the National Youth Policy Institute to secure basic data for establishing youth protection policies by understanding the current status of adolescents’ exposure to harmful environments in Korea. In basic research, the basic framework for the nature and content organization of integrated investigations was established. In order to faithfully achieve the purpose of the survey, which is to be used as basic data for policy responses related to youth protection, the overall content of the raw data was organized in a way that increased the degree of policy adherence compared to the previous survey (2).

Applying probability sampling, the participants were extracted using multistage cluster sampling. Poststratification weights were calculated by considering the size of the population by gender according to 17 cities/provinces and types of schools. The participants were 11,354 middle and high school students. The statistical data were granted confidentiality according to the Statistics Act No. 33.

### 2.2. Measurements

In this study, the following variables were used among the questionnaire items surveyed among adolescents by the Ministry of Gender Equality and Family (2).

#### 2.2.1. General characteristics

Gender was determined by a choice of “1: male, 2: female.” The categories of “drinking experience,” “smoking experience,” and “e-cigarette smoking experience” were determined by a choice of “1: yes, 2: no.”

#### 2.2.2. Autonomous control ability

Autonomous control ability was assessed by six items: “I am controlled by other people,” “I have few opportunities to decide things on my own,” “I often have to do what other people tell me to do in everyday life,” “I can freely express my thoughts and opinions in general,” “I can decide how to live my life on my own,” and “when I do something, I often follow other people’s way of thinking and acting rather than following my own.” Scores were calculated according to the scale of “1: strongly disagree, 2: disagree, 3: agree, 4: strongly agree.” In this study, Cronbach’s alpha was 0.76.

#### 2.2.3. Relationships with family, friends, and school teachers

Relationships with family, friends, and school teachers were assessed by five items. The subjects answered a total of 15 questions, each with 5 questions in relation to family, friends, and school teachers. “They (ex, family, friends, or school teachers) make me feel I’m loved and being taken care of,” “They are willing to listen to my worries and concerns,” “I can completely rely on them,” “They always pay attention to me and worry about me,” and “When I’m reluctant to make a decision, they would encourage me and reassure me.” Scores were calculated on a scale of 1: strongly disagree, 2: disagree, 3: agree, 4: strongly agree. In this study, Cronbach’s alpha was 0.95 for family relationships, 0.95 for friend relationships, and 0.96 for school teacher relationships.

#### 2.2.4. Experience of violence

Experience of violence was assessed by the following items: “I constantly hear curses or demeaning words targeted at me,” “I have been injured by hitting and kicking or by use of an object,” “Money or my other possessions have been taken from me,” “I have been bullied,” “I have been forced to do other’s chores,” and “I have been a victim of cyber bullying.” Each item was answered either “1: yes, 2: no.” Scores were calculated according to “1: yes, 0: no.”

#### 2.2.5. Experience of exposure to harmful media

Experience of exposure to harmful media was measured by a total of five questions; two asked whether the participants had watched R-rated adult videos or magazines in the past year, and three asked whether the participants had used new or variations of harmful media such as adult online games, gambling games involving betting money or cyber money, and messengers or chat apps for conditional dating in the past year. Each question was answered either “1: yes, 2: no.” Scores were calculated according to “1: yes, 0: no.”

#### 2.2.6. Runaway experience

Runaway experience was measured by responding “1: yes, 2: no” to “presence of runaway experience in the past year.” Runaway is defined as when a youth leaves home without the consent of a parent or guardian and does not return home for more than 24 h during the past year. The frequency of runaway experience was measured according to “1: none, 2: once, 3: twice or more.”

### 2.3. Data analysis

As the survey statistics data were collected by complex sampling design, complex sampling analysis was used to obtain the results. For the number of samples for each variable by item, the actual sample numbers from the raw data used in this study’s statistical analysis were used. Data were analyzed using the Statistical Package for Social Sciences IBM (SPSS-IBM), version 24 (SPSS, Inc., Chicago, Illinois, United States). The general characteristics of the variables were analyzed using descriptive statistics (frequency, percentage, mean, standard deviation). Differences in runaway experience according to general characteristics and related variables were analyzed by the *x*^2^ test and Fisher’s exact test for the categorical variables and a t-test for the continuous variables. Hierarchical logistic regression was used to determine the factors associated with the runaway experience of adolescents. The maximum value of variance inflation factor (VIF) between independent variables was 1.61, which was far below 10, and the minimum value of tolerance was 0.62, far above 0.20. Hence, there was no issue with multicollinearity. Obtaining informed consent was exempted by the Institutional Review Board (IRB) of Chosun University (IRB no. 2-1041055-AB-N-01-2019-34) because data were from the Comprehensive Survey of Adolescents’ Contact with Media Usage and Harmful Environment.

## 3. Results

### 3.1. General characteristics according to runaway experience

Analysis of differences in general characteristics according to runaway experiences revealed statistically significant differences in gender (*χ*^2^ = 11.09, *p* < 0.001), age (*χ*^2^ = 7.62, *p* = 0.006), drinking (*χ*^2^ = 66.03, *p* < 0.001), smoking (*χ*^2^ = 86.19, *p* < 0.001), and e-cigarette smoking (*χ*^2^ = 72.73, *p* < 0.001) according to runaway experience (Table 1).

**Table 1:** General characteristics according to runaway experience (*N* = 11,354).

Variables	Categories	Total	Runaway experience		*x*^2^*(*p*)
			Yes	No	
		*n* (%*)	*n* (%*)	*n* (%*)	
Gender^†^	Male	5,583 (52.3)	207 (3.7)	5,376 (96.3)	11.09 (<0.001)
	Female	5,087 (47.7)	133 (2.6)	4,954 (97.4)	
Age (year)^‡^	13–16	5,117(48.0)	184(3.6)	4,933(96.4)	7.62 (0.006)
	17–20	5,544(52.0)	154(2.8)	5,390(97.2)	
Drinking^§^	Yes	3,739(35.1)	211 (5.6)	3,528 (94.4)	66.03 (<0.001)
	No	6,918 (64.9)	129 (1.9)	6,789 (98.1)	
Smoking^ǁ^	Yes	1,224 (11.5)	112 (9.1)	1,112 (90.9)	86.19 (<0.001)
	No	9,444 (88.5)	228 (2.4)	9,216 (97.6)	
Electronic cigarette^¶^	Yes	848 (8.0)	83 (9.8)	765 (90.2)	72.73 (<0.001)
	No	9,818 (92.0)	257 (2.6)	9,561 (97.4)	

* Calculated by complex sample analysis.

† Skipped responses were excluded (*n* = 10,869).

‡ Skipped responses were excluded (*n* = 10,861).

§ Skipped responses were excluded (*n* = 10,857).

ǁ Skipped responses were excluded (*n* = 10,865).

¶ Skipped responses were excluded (*n* = 10,864).

### 3.2. Frequency of runaway experience according to general characteristics

Analysis of differences in the frequency of runaway experience according to general characteristics revealed statistically significant differences in gender (*χ*^2^ = 11.00, *p = 0*.004), age (*χ*^2^ = 6.00, *p* = 0.049), drinking (*χ*^2^ = 94.89, *p* < 0.001), smoking (*χ*^2^ = 138.58, *p* < 0.001), and e-cigarette smoking (*χ*^2^ = 111.69, *p* < 0.001).

The runaway experience was higher in males with 2.1% “once” and 1.6% “twice or more” than in females with 1.5% “once” and 1.1% “twice or more.” It was significantly higher in those aged 13–16 years with 1.9% “once” and 1.7% “twice or more” than for those aged 17–20 with 1.7% “once” and 1.1% “twice or more.” The frequency of runaway experience was significantly higher in those who drank alcohol with 3.2% “once” and 2.4% “twice or more” than in non-drinkers with 1.1% “once” and 0.8% “twice or more”; in smokers with 4.0% “once” and 5.2% “twice or more” than in non-smokers with 1.5% “once” and 0.9% “twice or more”; and in e-cigarette smokers with 3.8% “once” and 6.0% “twice or more” than those in e-cigarette non-smokers with 1.6% “once” and 1.0% “twice or more” (Table 2).

**Table 2:** Frequency of runaway experience according to general characteristics (*N* = 11,354).

Variables	Categories	Total	Runaway frequency			x* (*p*)
			Never	Once	Twice or more	
		*n* (%*)	*n* (%*)	*n* (%*)	*n* (%*)	
Gender	Male	5,583 (52.3)	5,376 (96.3)	118 (2.1)	89 (1.6)	11.00 (0.004)
	Female	5,087 (47.7)	4,954 (97.4)	76 (1.5)	57 (1.1)	
Age (year)	13–16	5,117 (48.0)	4,933(96.4)	98 (1.9)	86 (1.7)	6.00 (0.049)
	17–20	5,544 (52.0)	5,390(97.2)	94 (1.7)	60 (1.1)	
Drinking	Yes	3,739 (35.1)	3,528 (94.4)	120 (3.2)	90 (2.4)	94.89 (<0.001)
	No	6,918 (64.9)	6,789 (98.1)	73 (1.1)	56 (0.8)	
Smoking	yes	1,224 (11.5)	1,112 (90.9)	49 (4.0)	63 (5.2)	138.58 (<0.001)
	no	9,444 (88.5)	9,216 (97.6)	145 (1.5)	83 (0.9)	
Electronic cigarette	Yes	848 (8.0)	765 (90.2)	32 (3.8)	51 (6.0)	111.69 (<0.001)
	No	9,818 (92.0)	9,561 (97.4)	161 (1.6)	95 (1.0)	

*Calculated by complex sample analysis.

### 3.3. Differences in related variables according to runaway experience

Among the participants, 338 had runaway experiences. The mean value of their autonomous control ability was 2.99 ± 0.01, relationship with friends was 3.21 ± 0.01, relationship with family was 3.33 ± 0.01, and relationship with teachers was 2.99 ± 0.02. Experience of violence was 0.14 ± 0.01, and the experience of exposure to harmful media was 0.99 ± 0.02. Analysis of differences in related variables according to runaway experience indicated that autonomous control ability was significantly higher in the non-runaway group (2.99 ± 0.01) than in the runaway group (2.81 ± 0.03; *t* = −7.35, *p* < 0.001).

Relationships with friends (*t* = −2.35, *p* = 0.023), relationships with family (*t* = −10.38, *p < 0*.001), and relationships with teachers (*t* = −2.53, *p = 0*.015) were significantly lower in the runaway group than in the non-runaway group. Experience of violence (*t* = 6.09, *p* < 0.001) and exposure to harmful media (*t* = 7.32, *p < 0*.001) were significantly higher in the runaway group than in the non-runaway group (Table 3).

**Table 3:** Research variables according to adolescents’ runaway experiences (*N* = 11,354).

Variables	Total sample (*n* = 11,354)	Runaway*		t	*p*
		Yes (*n* = 338)	No (*n* = 10,531)		
	M ± SD	M ± SD	M ± SD		
Autonomous control ability	2.99 ± 0.01	2.81 ± 0.03	2.99 ± 0.01	−7.35	<0.001
Relationship with friends	3.21 ± 0.01	3.08 ± 0.05	3.21 ± 0.01	−2.35	0.023
Relationship with family	3.33 ± 0.01	2.94 ± 0.04	3.34 ± 0.02	−10.38	<0.001
Relationship with teacher	2.99 ± 0.02	2.85 ± 0.06	2.99 ± 0.02	−2.53	0.015
Experience of violence victimization	0.14 ± 0.01	0.46 ± 0.05	0.13 ± 0.01	6.09	<0.001
Harmful media	0.99 ± 0.02	1.53 ± 0.08	0.98 ± 0.02	7.32	<0.001

* Skipped responses were excluded (*n* = 10,869).

### 3.4. Factors influencing adolescents’ runaway experience

For Model 1, hierarchical logistic regression was performed by introducing demographic characteristics that displayed significant differences in univariate analysis to determine the factors associated with adolescents’ runaway experiences.

Model 1 indicated that age, drinking, and smoking are predictors influencing runaway experience and the regression model was significant (Wald *χ*^2^ = 306.82, *p* < 0.001). The Cox and Snell R^2^ value was 0.019 and the Nagelkerke R^2^ value was 0.078. For Model 2, autonomous control ability, relationship with friends, relationship with family, relationship with teachers, the experience of violence, and experience of exposure to harmful media were added. Model 2 revealed that the independent variables that were associated in Model 1 were significant. They were also predicted by an autonomous control ability, relationship with family, the experience of violence, and experience of exposure to harmful contents, and the regression model was significant (Wald *χ*^2^ = 766.44, *p* < 0.001). The Cox and Snell R^2^ value was 0.032, and the Nagelkerke R^2^ value was 0.131 (Table 4). Those aged 13–16 years had increased runaway experience compared to those aged 17–20 years by 2.20 (95% Cl 1.75–2.77). Drinking showed increased runaway experience compared to non-drinking by 2.19 (95% Cl 1.73–2.78), and smoking showed increased runaway experience compared to non-smoking by 2.00 (95% Cl 1.38–2.89).

**Table 4:** Factors influencing adolescents’ runaway experience (*N* = 11,354).

Categories (reference)		Model 1^†^						Model 2^‡^					
		B	S.E	OR	95%CI		*p*	B	S.E	OR	95% CI		*p*
					Low	High					Low	High	
Constant		−4.507	0.157	-	-	-	<0.001	−2.426	0.396				<0.001
Gender	Female	Reference											
	Male	−0.054	0.120	0.95	0.75	1.20	0.654	−0.018	0.157	0.98	0.72	1.34	0.908
Age (year)	17–20	Reference											
	13–16	0.783	0.129	2.19	1.70	2.82	<0.001	0.789	0.117	2.20	1.75	2.77	<0.001
Drinking	No	Reference											
	Yes	1.006	0.122	2.74	2.15	3.47	<0.001	0.785	0.122	2.19	1.73	2.78	<0.001
Smoking	No	Reference											
	Yes	0.809	0.184	2.25	1.57	3.23	<0.001	0.691	0.189	2.00	1.38	2.89	<0.001
Electronic cigarette	No	Reference											
	Yes	0.468	0.248	1.60	0.98	2.60	0.06	0.416	0.241	1.52	0.95	2.43	0.085
Autonomous control ability								−0.276	0.107	0.76	0.62	0.94	0.010
Relationship with friends								0.221	0.195	1.25	0.85	1.83	0.258
Relationship with family								−0.730	0.086	0.48	0.41	0.57	<0.001
Relationship with teacher								0.044	0.163	1.04	0.76	1.44	0.790
Experience of violence victimization								0.256	0.052	1.29	1.17	1.43	<0.001
Experience of harmful media								0.209	0.057	1.23	1.10	1.38	<0.001
Wald $\chi^{2}$ (*p*)		306.82 (<0.001)						766.44(<0.001)					
Cox and Snell R^2^		0.019						0.032					
Nagelkerke R^2^		0.078						0.131					
C		0.704						0.774					

† Skipped responses were excluded (*n* = 10,789).

‡ Skipped responses were excluded (*n* = 10,783).

^*^OR, Odds ratio; CI, Confidence interval; C, concordance index.

Higher autonomous control ability showed decreased runaway experience by 0.76 (95% Cl 0.62–0.94), while a higher level of positive perception toward family relationships showed decreased runaway experience by 0.48 (95% Cl 0.41–0.57). Increasing experience of violence showed an increase in runaway experience by 1.29 (95% Cl 1.17–1.43) and increasing experience of exposure to harmful media showed an increase in runaway experience by 1.23 (95% Cl 1.10–1.38; Table 4).

## 4. Discussion

### 4.1. Characteristics of adolescents according to runaway experience

First, among the general characteristics, adolescents’ runaway experiences according to individual factors showed differences in gender and age. Studies (3, 25, 26, 43) have reported more runaway experiences in female adolescents.

However, there was a difference in our results as male adolescents ran away from home more than female. The results of this study agree that gender is a significant variable, but which gender is higher in runaways and frequency differs from previous studies. Whether other characteristics affect the result in association with gender difference needs to be further examined through objective and comprehensive replication studies. Furthermore, differential strategies according to gender characteristics should be established. Lee (29) and Oh (26) determined age to be an influencing factor of runaway experience, showing results similar to this study. However, Kim (27) showed no difference in runaway experience between middle school and high school students, which is in contrast to this study’s results. However, a decrease in starting age for adolescents’ runaway behavior (1) as well as an increase in runaway frequency was verified in this study. As a runaway experience at a younger age can increase the number of runaway experiences, proper and early intervention is required.

Next, this study’s results indicated that drinking, smoking, and e-cigarette smoking were linked to differences in runaway experiences. This is in line with Seng (44), who reported that runaway adolescents indulge in drinking and smoking the most.

Analyzing differences in the degrees of related variables according to runaway experience showed differences according to autonomous control ability, relationship with friends, relationship with family, relationship with teachers, the experience of violence, and the experience of exposure to harmful media.

### 4.2. The complex predictors in runaway behavior among youth

To determine the variables that predict runaway experience among those that exhibited differences in the univariate analysis, hierarchical logistic regression was performed by introducing individual characteristics, autonomous control ability, the experience of violence, and experience of exposure to harmful media through two stages.

The level of influence by outcome variables could be compared according to the stage. In the first stage, age, drinking, and smoking, which are individual characteristics, were determined as variables affecting the runaway experience. In the second stage, age, drinking, smoking, autonomous control ability, relationship with family, the experience of violence, and the experience of exposure to harmful media were determined as significant variables affecting runaway experience. The explanatory power was 7.8% when only individual characteristics were introduced, but it increased to 13.1% when related variables were additionally introduced.

Runaway experience increased in those aged 13–16 compared to those aged 17–20 with an odds ratio of 2.20, and drinkers and smokers showed increased runaway experience with an odds ratio of 2.19 and 2.00, respectively. Drinking and smoking, which are delinquent behaviors, influence runaway experiences. Past 12-month alcohol use, and past 30-day cigarette use were all associated with higher odds of running away from home (45), which is consistent with the findings of this study.

Establishing peer groups with adolescents who are engaged in drinking and smoking can lead to runaway behavior and the formation of a runaway family, which can ultimately lead to greater negative consequences (16). It has been reported that interaction with antisocial peers can predict homelessness (31). Therefore, to prevent deviant behavior that can occur in a complex manner emotional support and early intervention are needed for those who already have runaway experiences, in addition to prevention education for smoking and drinking.

Autonomous control ability was determined as an influencing factor for runaway experience as higher autonomous control ability led to a decrease in runaway experience with an odds ratio of 0.76. It has been reported that low autonomous control ability leads to a higher probability of deviant behavior (46), which supports this study’s results. It is also consistent with research showing that low levels of self-control are associated with antisocial outcomes (40). Autonomy is the ability to manage and control oneself. Improving and efficiently using autonomous control ability, which allows one to restrict and control one’s behavior when exposed to problematic circumstances, will enable one to control one’s emotions and behavior and prevent deviance. It will also help establish one’s identity and restrict delinquent behavior such as running away from home.

Therefore, emotional support from family and school and the development of an intervention program are required.

Next, the family relationship was statistically significant as a variable affecting runaway behavior. Greater positive relationships with family led to a decrease in runaway behavior with an odds ratio of 0.48. The results of this study are consistent with reports that supportive relationships in families influence runaway behavior among family factors (24). Early adolescents are vulnerable to family conflicts, which can increase the risk of running away from home. It also emphasizes the need to develop primary prevention programs that build healthy relationships between family members during adolescence (31). Numerous factors such as family support, structure, function, and economic characteristics serve as important variables for adolescents’ runaway experiences. Hence, reinforcing family functions such as parental roles is critical. To achieve this, understanding the family characteristics of adolescents, providing parent education programs, and systematic support for vulnerable families are required so that active intervention from parents can prevent their children’s runaway behavior. Although adolescents have a strong desire to gain independence from their parents, they need the emotional support of a system within a stable family structure.

Experience of violence was verified as an important factor influencing runaway experience as its increase led to increased runaway behavior with an odds ratio of 1.29. Compared to students living in stable homes, students experiencing homelessness were three times more likely to be threatened or injured with a weapon at school (10). This was supported by a few studies’ results (24, 26, 30), which reported that the frequency of runaway experiences increases upon experiencing school violence. The location of violence was mostly inside schools, and the adolescents who reported violence only accounted for 46.5% (2). To escape from violence, adolescents choose to run away from home, and this leads them to a harsher environment as returning home becomes a difficult option. Therefore, to reduce violence, regular counseling and management by school nurses and homeroom teachers, and active intervention by schools are required. They need to investigate violence inside a school, identify victims early, examine the damage, and help their recovery. An intervention program providing coping strategies is also needed.

Finally, the runaway incidents increased according to the experience of exposure to harmful media with an odds ratio of 1.23. As an influencing factor, harmful media exhibited differences according to each type, and this study validated that exposure to harmful media can predict runaway experiences. The environment people live in has been rapidly changing recently due to the prodigious development of media-related technology. Internet and smartphone usage has rapidly increased among adolescents, which has led to the possibility of their exposure to harmful media and the risk of committing sex crimes. Therefore, understanding the characteristics of various types of harmful media and the diagnosis of problems is required (22, 47). For adolescents, who are particularly sensitive to new technology and media use, smartphones have become an important part of their lives (48) as 80.7% of teenagers use the Internet/mobile messenger almost every day, and a 20-year survey showed a steady increase in the number of adult videos marked as not available for adolescents to watch (1).

Prevention programs for internet addiction according to age need to be established. The experience rate of conditional encounter messenger or chat apps is 3.8%, which is slightly higher than in 2016 (1). It is imperative to carefully examine the factors that increase the rate of exposure to new variants of harmful media and prepare countermeasures accordingly.

Therefore, school nurses should include adverse effects of harmful content and preventive management in education programs. In the school setting, there is an urgent need to educate adolescents on harmful media. Health education programs customized for each gender, grade level, and local characteristics, according to their level of internet addiction and the addicted subject, need to be reinforced, so that adolescents recognize the serious risk and negative effects of constant exposure to various harmful media, and are no longer exposed to them.

The uses and gratifications theory suggests that social media (e.g., smartphones) are often used to fulfill one’s unmet needs (49). Positive links were identified between social media and risky behaviors during adolescence in this meta-analysis (50). Hwang et al. (47) found that unsatisfactory family and school environments lead to higher usage of harmful media. This suggests that each variable of family, society, and environment, in addition to individual characteristics, leads to an increased risk of runaway behavior in combination. Therefore, various approaches from an ecological perspective, as well as proper management and regulation, are required for adolescents to comfortably thrive in a healthy media environment.

### 4.3. Limitations

This study used basic data provided by highly representative raw national data obtained from a nationwide complete enumeration survey on adolescents. However, secondary analysis of the raw data does not allow the use of various related variables based on the literature and cannot change the variables investigated. This study failed to measure related variables in the utilization of development tools whose reliability and validity have been verified. Due to the limitations of the variables, the explanatory power of this study is somewhat low. Juvenile runaway behavior is caused by complex and dynamic processes, so it is necessary to investigate the causes of runaway behavior from multiple perspectives. In this context, this study holds significance as it revealed influencing factors from multilateral aspects of individual, family, school, society, and environmental variables instead of one factor.

Since 2016, the media variable has been included in the harmful environment survey for the first time in South Korea. The 2016 data was analyzed because it was the first meaningful data to include media in the analysis. It is suggested to analyze the longitudinal influence of media on runaways based on the results of media research analysis in 2016 as basic data.

## 5. Conclusion

Adolescents develop in diverse and complex environmental systems, and individual adolescents are considered part of these systems. In order to minimize youth exposure to harmful environments, social cooperation systems such as school environments, homes, communities, health centers, and social workers should be established to evaluate and mediate the school environment. Acquiring knowledge of the initial data on the community-based runaway incidence and risk factors can provide information to understand the temporal trends of runaway incidence and risk factors in South Korea. This study also empirically validated that harmful media that have recently become diverse and accessible can influence adolescents to run away from home.

This study is also meaningful from the nursing aspect as it highlights the need for health education reinforcement for reducing adolescents’ exposure to harmful media. The association of adolescents’ runaway experience and emotional impairment such as depression and impulsiveness as individual characteristics, in addition to behavioral factors, needs to be clarified. Moreover, various factors that can affect adolescents’ runaway behavior should be further investigated with the inclusion of a wider range of variables. Further studies are also needed on developing early intervention programs that can prevent runaway behavior.

## Data availability statement

Publicly available datasets were analyzed in this study. Data are available by request to the Ministry of Gender Equality and Family and National Youth Policy Institute.

## Ethics statement

The requirement of ethical approval was waived by Institutional Review Board (IRB) of Chosun University for the studies involving humans because data were from the Korean Survey of (Comprehensive Survey of Adolescents’ Contact with Media Usage and Harmful Environment). The studies were conducted in accordance with the local legislation and institutional requirements. Written informed consent for participation was not required from the participants or the participants’ legal guardians/next of kin because This study is a secondary data analysis of 2016 Comprehensive survey of adolescents’ contact with media usage and harmful environment.

## Author contributions

HRK: conceptualization, methodology, data analysis, and writing—original draft preparation. S-HM: conceptualization, methodology, writing—review and editing, and project administration. All authors contributed to the article and approved the submitted version.

## Funding

This study was supported by research fund from Chosun University, 2019.

## Conflict of interest

The authors declare that the research was conducted in the absence of any commercial or financial relationships that could be construed as a potential conflict of interest.

## Publisher’s note

All claims expressed in this article are solely those of the authors and do not necessarily represent those of their affiliated organizations, or those of the publisher, the editors and the reviewers. Any product that may be evaluated in this article, or claim that may be made by its manufacturer, is not guaranteed or endorsed by the publisher.
